# Fractured coracoid process with acromioclavicular joint dislocation: A case report

**DOI:** 10.1097/MD.0000000000022324

**Published:** 2020-09-25

**Authors:** Wei Zhang, Bingzhe Huang, Jingjing Yang, Pan Xue, Xiaoning Liu

**Affiliations:** Orthopaedic Medical Center, the Second Hospital of Jilin University, Changchun, China.

**Keywords:** acromioclavicular dislocation, coracoid processes, fracture

## Abstract

**Rationale:**

Coracoid processes (CPs) fracture with acromioclavicular (AC) joint dislocation are extremely rare. This combined injury has brought many challenges to surgeons, and the mechanism underlying the injury is still not fully understood. There is no clear consensus on its treatment.

**Patient concerns:**

Here, we describe a CP fracture with AC joint dislocation in a middle-aged manual worker.

**Diagnosis:**

Radiographs showed a fracture of the base of the CP and a third-degree AC joint separation.

**Interventions:**

The patient was treated surgically with open reduction and internal fixation of the AC joint by LCP clavicle hook plate, and the CP was fixed with a 3.5 mm diameter cannulated screw.

**Outcomes:**

Three months after the operation, shoulder function was completely restored, and the affected shoulder had full mobility with no tenderness. Plain film radiography showed anatomical indications of the healing of these combined injuries.

**Lessons:**

Although AC joint dislocation with CP fractures is extremely rare in adults, it is important to remind and remember that this possibility exists. In unclear cases, special radiographic films and CT are necessary. Surgical treatment of AC joint dislocation with CP fractures can provide solid stability and restore normal shoulder function with an excellent prognosis.

## 1 Introduction

The incidence of scapular fractures is not high, accounting for only 1% of all fractures, and fractures of the coracoid processes (CPs) are also relatively rare, accounting for 3% to 13% of scapular fractures.^[1–3]^ CP fractures with acromioclavicular (AC) joint dislocation are even rarer. There are only a few cases reported in the literature.^[3–6]^ This combined injury has brought many challenges to surgeons, and the mechanism underlying the injury is still not fully understood. There is no clear consensus on its treatment.

In the present case, we describe a CP fracture with AC joint dislocation in a middle-aged manual worker. Patients and their families have provided written consent to publish the case report, and the institutional review board of the Second Hospital of Jilin University approved the study.

## 2 Case report

**Figure 1:**
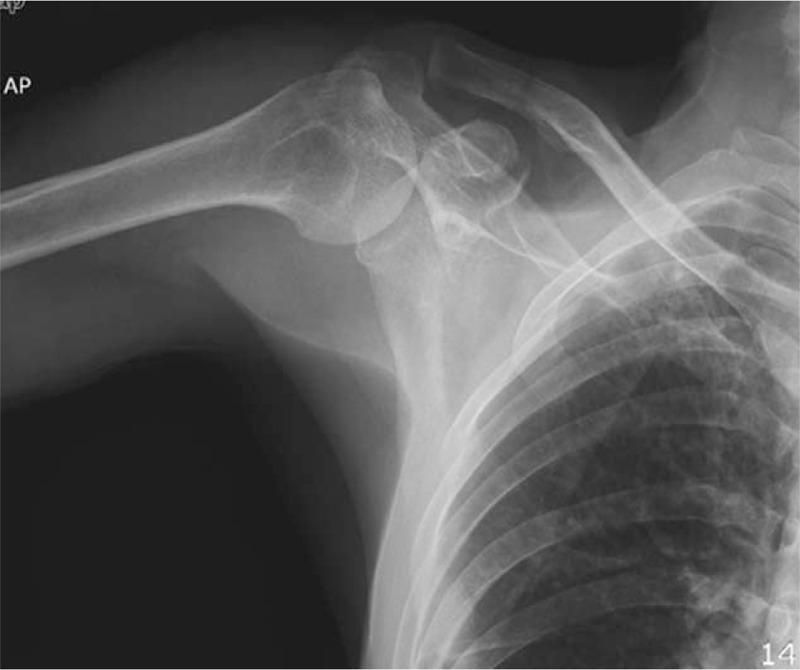
Preoperative anteroposterior X-ray showed CP fracture and a third-degree AC joint separation.

**Figure 2:**
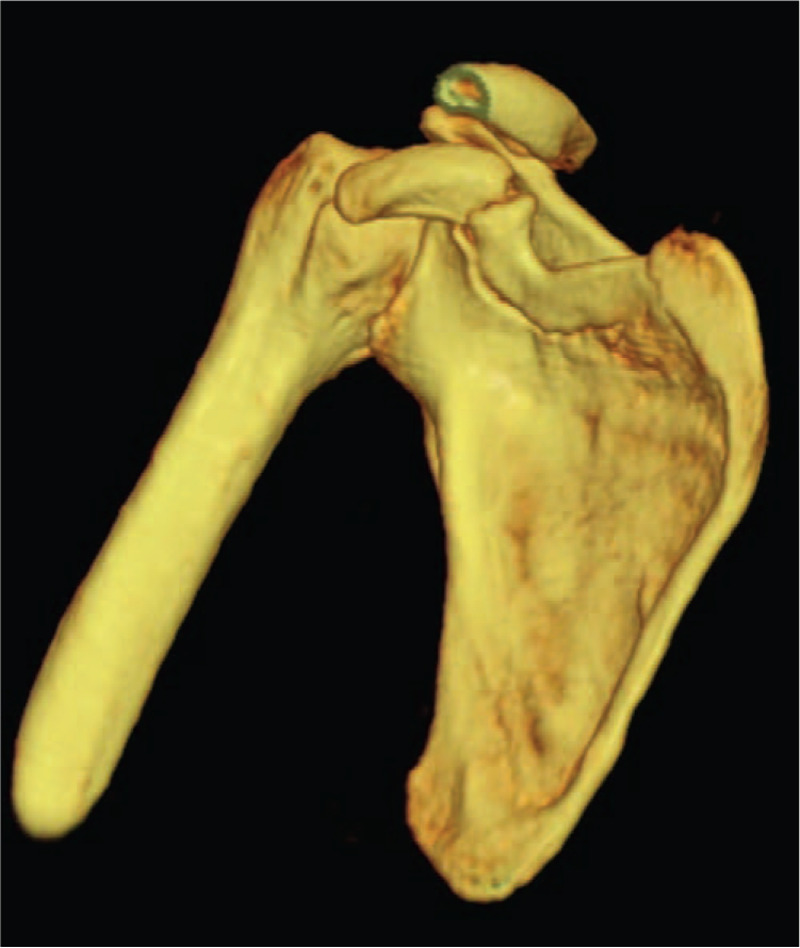
Preoperative 3D-CT showed a displaced fracture of the coracoid process base.

A 55-year-old worker suffered from trauma to his right shoulder after he fell from a bicycle due to a car accident and landed on the right side of his body. He had a major injury and fractured multiple ribs. Physical examination showed a prominent distal clavicle and tenderness over the AC joint and the CP. The range of motion of the right shoulder was decreased due to pain. The results of the neurovascular examination were essentially normal. Radiographs showed a fracture of the base of the CP and a third-degree AC joint separation (Figs. 1 and 2).

**Figure 3:**
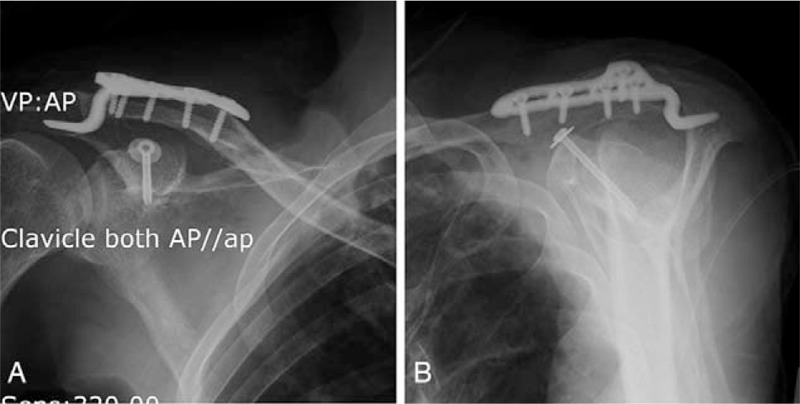
AC joint was fixed by LCP clavicle hook plate; the CP was fixed with a 3.5 mm diameter cannulated screw. Immediate postoperative anteroposterior X-ray view (A), immediate postoperative outlet X-ray view (B).

He was treated surgically with open reduction and internal fixation of the AC joint by LCP clavicle hook plate (Synthes Inc, PA, US). The CP was fixed with a 3.5 mm diameter cannulated screw (Synthes Cannulated Screw System, Synthes Inc., PA) (Fig. 3). The positions of the CP and the screw were confirmed with intraoperative radiographic screening. The patient had an uneventful postoperative course and was discharged on the fifth day with an arm sling.

The patient's shoulder was placed in a sling after surgery, and the pendulum movement began immediately after surgery. The patient was advised not to engage in elbow flexion or place any weight on that arm for 6 weeks after surgery. The range of motion of the shoulder was limited to 45° abduction and 90° forward flexion. After 6 weeks, the patient was permitted to begin progressive active activities. Muscle exercises began at 8 weeks.

Three months after the operation, shoulder function was completely restored, and the affected shoulder had full mobility with no tenderness. Plain film radiography showed anatomical indications of the healing of these combined injuries.

## 3 Discussion

CP fractures with AC joint dislocations rarely occur in adults, and there is still disagreement about the underlying mechanisms.^[1,3,7]^ A direct blow with cephalad-to-caudad violence and alternatively a strong contraction of the short head of the biceps and pectoralis minor muscles are both plausible mechanisms. The sudden pull of the combined tendon and pectoralis minor could cause the CP to break. Whether the coracoclavicular ligament remains intact depends on the ligament's component residual force. In AC dislocations with CP fractures but intact coracoclavicular ligaments, as in our patient, CP fractures are often associated with type 3 AC dislocations.

In such cases of combined injuries, if only conventional radiography is used, AC joint dislocation is often pronounced, which draws considerable attention, and the CP fracture may be ignored. At this time, computed tomography (CT) is necessary to clearly show the images of CP fractures and AC joint dislocations to avoid misdiagnosis. For this reason, we recommend that, for patients with AC joint dissection and shoulder injuries, the anterior and posterior view of the shoulder joint, oblique position, axillary view is necessary.

Previously published case reports include both surgical and conservative treatment for this combined injury.^[3,5,8,9]^ Overall, more cases have been treated with surgery than with conservative treatment. As this type of combined injury is extremely rare, it is difficult to compare the advantages and disadvantages of the 2 treatments without a large cohort. According to the current literature, in these concomitant injuries, there are no differences in the long-term outcomes of patients treated conservatively and surgically.^[3,5,8,9]^ However, some earlier reports mentioned that AC joint pain and cosmetic symptoms persist after conservative treatment.^[10]^ This may be related to AC joint capsule entrapment and fibrocartilage disc rupture. For this combined injury, several surgical methods have been reported in the literature. Some people choose to only address the AC joint, while others have found that AC can be reduced indirectly after CP reduction by surgery.^[9,11]^ In this regard, we believe that in cases of patients who perform manual labor or heavy-duty work, it is more logical that both injuries (AC dislocation and CP fracture) should be fixed. For patients with lower functional requirements, it is feasible to deal with only 1 injury. Obtaining reliable stability for CP fractures and AC joint dislocations can help patients recover early. Considering that this case is a manual laborer and the double structure injury of the shoulder joint complex, we chose surgical treatment to obtain rigid fixation. In addition to the tension screws used for CP fractures, we also used the traditional method for fixing isolated AC joint dislocations, using a hook plate to fix the AC joint. This double fixation method can reduce the tensile force of the CC ligament on the CP, so that the 2 injuries can be healed early and function can be exercised early as well. At week 8 after surgery, the shoulder joint was painless, active, and strong.

## 4 Conclusion

Although AC joint dislocation with CP fractures is extremely rare in adults, it is important to remind and remember that this possibility exists. In unclear cases, special radiographic films and CT are necessary. Surgical treatment of AC joint dislocation with CP fractures can provide solid stability and restore normal shoulder function with an excellent prognosis.

## Author contributions

**Conceptualization:** Wei Zhang, Xiaoning Liu.

**Formal analysis:** Jingjing Yang.

**Funding acquisition:** Xiaoning Liu.

**Investigation:** Bingzhe Huang.

**Methodology:** Bingzhe Huang, Pan Xue.

**Writing – original draft:** Wei Zhang, Bingzhe Huang, Pan Xue, Xiaoning Liu.

**Writing – review & editing:** Jingjing Yang, Xiaoning Liu.
